# Assessment of primary care facilities for cardiovascular disease preparedness in Madhya Pradesh, India

**DOI:** 10.1186/s12913-015-1075-x

**Published:** 2015-09-23

**Authors:** Abhijit Pakhare, Sanjeev Kumar, Swati Goyal, Rajnish Joshi

**Affiliations:** Department of Community and Family Medicine, All India Institute of Medical Sciences Bhopal, Bhopal, India; Gandhi Medical College, Bhopal, India; Department of General Medicine, All India Institute of Medical Sciences Bhopal, Bhopal, India

## Abstract

**Background:**

Government of India has launched National Program for Prevention & Control of Cancer, Diabetes, Cardiovascular Diseases and Stroke (NPCDCS) to address high prevalence of non-communicable diseases (NCDs) in India. Cardiovascular diseases (CVDs) constitute a significant portion of NCD burden. While this program is yet to be launched in all districts of Madhya Pradesh state of India, we performed this study to understand facility-level gaps that need to be addressed to improve CVD services in primary care provided by the public sector.

**Methods:**

This is a cross-sectional questionnaire based study. A standardized questionnaire was self-administered to 85 medical officers from as many primary care facilities from 24 districts of the state. These medical officers were working in two types of primary care facilities – primary health center (PHC) and community health centers (CHC). Facilities were assessed for 36 items in 5 domains (human-resource, equipment, drug supplies, point-of-care tests and laboratory services) with a focus on management of hypertension and diabetes mellitus in primary-care. Each item was to be answered as either present or absent at the facility where medical officer was working. We compared availability of an item across two levels of primary care facilities. All statistical analysis were done using Microsoft Excel.

**Results:**

Availability of facilities was least in laboratory services, and human resource domains followed by drugs, and better in equipment and point-of-care supply domains. Across these domains, availability of items in CHCs was (37.1, 49.0, 56.1, 67.9 and 80.9 % respectively) and in PHCs (11.8, 18.2, 44.2, 55.1, and 55.3 % respectively).

**Discussion:**

Current facility assessment study shows critical gaps in key items required for management of NCDs at primary care level. Human resource and laboratory services need to be strengthened the most, followed by sustained availability of all required drug classes, equipment and related supplies, and upgrading point-of-care testing. There are larger gaps in PHCs, which are level 1 facilities, as compared to CHCs, which are level 2 facilities in primary-care.

**Conclusions:**

Increasing burden of NCDs like hypertension and diabetes mellitus necessitates public health response through health systems. Therefore health system preparedness in form of trained human resources, functional laboratories and well stocked pharmacies are essential in primary care facilities.

## Background

Non-communicable diseases (NCDs), especially cardiovascular diseases (CVD; coronary artery, cerebro-vascular, and peripheral vascular diseases) are a leading cause of mortality among middle aged and older adults in India [1]. Prevalence of CVD risk factors (smoking, hypertension, diabetes mellitus, obesity, dyslipidemia, unhealthy diet, and reduced physical activity) is increasing, and recent studies report up to 40 % of Indian adults to be hypertensive [2], and 10 % to be diabetic [3]. Risk factor control requires a multidisciplinary approach, which includes approaching social determinants of health, health-care financing, improving medical education, and health system strengthening to better manage risk factors [4]. Except for smoking and low vegetable and fruit diet, most of the risk factors for CVD are more prevalent in high socioeconomic groups [5]. However considering large proportion of population in lower socioeconomic group, it has been suggested that most of the burden of risk factors is contributed by poor and underprivileged [6], which puts higher onus on public health delivery system for NCD care in India.

Government of India has responded to the increasing burden of NCDs through launch of a National Program for Prevention & Control of Diabetes, Cardiovascular Diseases and Stroke (NPCDCS) in 2008 [7]. Currently in its second phase, the program includes 100 of 640 districts (15.6 %) in India. The NPCDCS aims at integration of NCD interventions in the overall public-health delivery framework for optimization of scarce resources and provision of seamless services to patients as also for ensuring long term sustainability of interventions [7]. WHO developed a global monitoring framework to enable global tracking of progress in preventing and controlling major non-communicable diseases - cardiovascular disease, cancer, chronic lung diseases & diabetes and their key risk factors. Targets and indicators for availability of essential non-communicable disease medicines and basic technologies to treat major non-communicable diseases have been described under national systems response category. An 80 % availability of the affordable basic technologies and essential medicines, including generics, required to treat major non-communicable diseases in both public and private facilities is expected to be achieved by 2025. In this context Government of Madhya Pradesh has launched free drug distribution and laboratory investigation scheme in public health facilities. While this is a work in progress, and the program is likely to be scaled up to all the districts of the country, it is important to understand existing infrastructure and readiness of public health infrastructure for NCD care.

Public health delivery system in rural India consists of Primary Health Centers (PHC; n ~ 25,000; catchment population ~30,000) as a level 1 facility, and a Community Health Center (CHC; n ~ 5000; catchment population ~ 100,000) a level 2 facility. CHC functions as a level 1 facility for the village it is located-in, and level 2 facility for the villages catered by adjoining PHCs in the catchment area. Together, these facilities are designed to meet most primary care needs through medical doctors, paramedical staff, basic investigations and essential drugs [8]. Over the years, focus of primary care in India has been communicable diseases, and reproductive and child health services. As a consequence, it is likely that some key facility-based elements required for NCD-care may be lacking. The current study was performed to understand availability of health-facility level infrastructure for delivery of CVD care in PHCs and CHCs in state of Madhya Pradesh.

## Methods

### Setting

Madhya Pradesh is second largest state in India by area, and sixth by population (population ~ 72 million (2011 census)) and has network of 1157 PHCs and 334 CHCs [9]. Between May and August 2013, Department of Public Health and Family Welfare, Government of Madhya Pradesh, and All India Institute of Medical Sciences Bhopal conducted workshops for medical officers posted in PHCs and CHCs of the state. Purpose of these workshops was to upgrade skills with regards to identification and management of hypertension, diabetes and CVD risk factors. Request for nomination for these workshops were sent by Directorate Health Services to Chief Medical Officers of 24 districts. These districts have a network of 169 CHCs and 640 PHCs. Nominations of 66 and 56 medical officers of PHCs and CHCs were received of which 48 and 39 attended the workshops respectively. These Medical officers belonged to five districts where NPCDCS has been launched in the state (Chindwara, Dhar, Hoshangabad, Jhabua and Ratlam) and another nineteen districts (Balaghat, Betul, Bhind, Bhopal, Dindori, Gwalior, Khandwa, Khargone, Mandsaur, Morena, Raisen, Satna, Sehore, Seoni, Shahdol, Shajapur, Shivpuri, Sidhi, and Ujjain). We performed a questionnaire-based survey to understand facility level infrastructure in the State.

### Participants

A total of 85 (97.7 %) Medical officers (38 from CHC and 47 from PHCs) participated in the survey and provided facility level information. Workshop was conducted in batches, with a maximum batch size of 20. A total of five workshops were conducted, and questionnaire was administered at the start of each workshop, and participation was voluntary. In order to ensure a correct reporting, and to protect participants from potential administrative repercussions on reporting of deficiencies in the system, we decided not to collect any personal identifiers (Name of respondent, or the facility) for this study. Hence we requested Institutional Human Ethics Committee (IHEC) of AIIMS Bhopal for a waiver of consent. This request and the study design were approved by the IHEC of AIIMS Bhopal.

### Procedure

We designed a facility assessment questionnaire based on Indian Public Health Standards (IPHS) for PHC and CHC [10], and World Health Organization package of essential NCD interventions (WHO PEN) guidelines [11]. We initially consulted core team of medical and public health specialists (who were also involved in the designing training curriculum for the medical officers), to draw up a list of tools and technologies required for primary care management of key NCDs. Items in this list that were also included in either WHO-PEN or IPHS were retained. World Health Organization has proposed a package of essential NCD interventions (WHO PEN) for low resource settings [11]. This package lists simple technologies that must be available, and others to be added when resources permit. While WHO-PEN provides for a list of essential technologies and drugs required in primary care, it neither details human resource requirements, nor does it discriminate between levels of Primary care. IPHS has separate standards for PHC & CHC, including human resources, infrastructure, equipment & drugs, but does not have a NCD focus. We took most items from the PEN and relevant items from IPHS standards in the current study. The complete set of expanded questionnaire with source of individual items is presented in Table 1. We divided all the items of the questionnaire in five domains (Human resources, equipment, point-of-care supplies, laboratory based tests and drugs), and for two disease conditions (hypertension and diabetes). We included four basic drugs needed in management of hypertension (Angiotensinin Converting Enzyme Inhibitors (ACEI)/ Angiotensin receptor blockers (ARB), Beta blockers, Calcium channel blockers, (CCB) and Thiazide diuretics) and four drugs commonly used in management of diabetes mellitus (Metformin, Glibenclamide, Glimiperide, and Insulin) in the questionnaire. In each domain, we provided a list of facilities, and expected answer was to be either Yes or No or do-not know (coded as 1, 0 and 99 respectively). We grouped key facilities as needed in management of diabetes and hypertension to understand preparedness for their management in public health system. There were two versions of the questionnaire; first version with 15 items was administered to 48 medical officers. We expanded the questionnaire in later part of the study, largely to assess for availability of essential drugs and medications pertaining to NCDs. The final version was expanded to include 36 items, which was administered to 37 medical officers who reported in remaining batches.

**Table 1:** Domains, sub-groups and key items evaluated in the questionnaire

Domain	Sub-group	Key items evaluated	Source of item
Human resource		Two Medical Officers	IPHS-PHC & CHC
		Laboratory technician	IPHS-PHC & CHC
		Health Educator	IPHS-PHC & CHC
		Nutritionist	IPHS-CHC (Dietician)
		Physiotherapist	IPHS-CHC (Rehabilitation worker)
Equipment	Equipment for diagnosis of hypertension	Sphygmomanometer (Mercury or Aneroid)^a^	IPHS-PHC & CHC,PEN
	Equipment for diagnosis of diabetes	Glucometer^b^	PEN
	Equipment for evaluation of neuropathy	Percussion Hammer or Tuning fork	IPHS-PHC & CHC,PEN (Only tuning fork)
	Equipment for assessment of abdominal obesity	Measuring tape	PEN
	Equipment to measure body mass index (BMI)	Stadiometer and weight machine	IPHS-PHC & CHC, PEN (Only weight machine)
	Equipment to evaluate cardiac complication	ECG machine^a^	IPHS-CHC, PEN
Point-of-care testing supplies		Glucose testing strips	PEN
		Lancets/needle for finger-prick	PEN
		Urinary strip for Ketones^b^	PEN
		Urinary strip for Proteins^ba^	PEN
Laboratory service		Plasma Glucose estimation^b^	PEN
		Serum creatinine^ba^	IPHS-CHC, PEN
		Serum lipid profile	IPHS-CHC, PEN
		HbA1C estimation	None
Drugs	Drug useful for long term management of Hypertension and Lipid lowering drugs^a^	ACEI/ARB (Enalapril, Ramipril, Losartan etc.)	IPHS-PHC & CHC, PEN
		Beta blocker (Atenolol)	IPHS-PHC & CHC, PEN
		CCB (Amlodepine)	IPHS-PHC & CHC, PEN
		Diuretic (Thiazide)	IPHS-PHC & CHC, PEN
		Lipid lowering (Atorvastatin)	IPHS-PHC & CHC, PEN
	Drug useful for long term management of Diabetes MellitusL^b^	Metformin	IPHS-PHC & CHC, PEN
		Glibenclamide	IPHS-PHC & CHC, PEN
		Glimepiride	IPHS-PHC & CHC, PEN
		Pioglitazone	IPHS-PHC & CHC, PEN
		Insulin (any)	IPHS-PHC & CHC, PEN
	Drugs used in management of chronic disease emergencies and Dexamethasone, which is frequently misused	Aspirin low dose	IPHS-PHC & CHC, PEN
		Nitrate (Sorbitrate)	IPHS-PHC & CHC, PEN
		Nifedepine	IPHS-PHC & CHC, PEN
		Frusemide	IPHS-PHC & CHC, PEN
		Dexamethasone	IPHS-PHC & CHC

^a^These facilities are key for primary care management of hypertension

^b^These facilities are key for primary care management of diabetes mellitus

*IPHS* Indian Public Health Standards (for *PHC* (Primary health centers 2012) or *CHC* (Community health centers 2012)), *PEN* Package of essential non-communicable disease interventions for primary care

### Statistical analysis

We performed descriptive analysis for the collected data and analyzed frequencies separately for community health centers (a facility with higher level of functionality) and primary health centers (a facility with a lower level of functionality). We averaged proportions for individual items under a domain, to indicate coverage in a particular domain. For instance, to indicate overall drug availability, we averaged proportions of all drug classes included in the survey. To estimate readiness for individual diseases such as hypertension or diabetes mellitus, we used disease-specific indicators in point-of-care tests, laboratory tests, and drug domains. Statistical analysis was performed in Microsoft Excel version 2010.

## Results

**Fig. 1:**
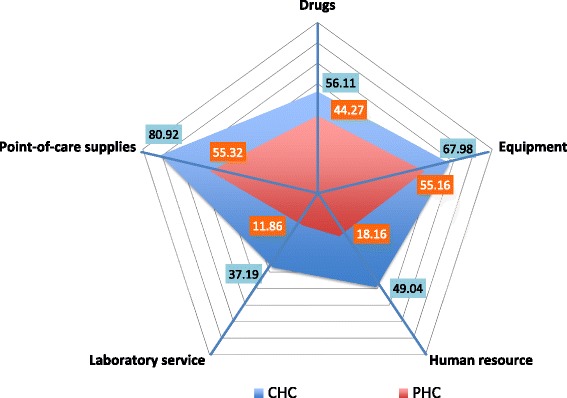
Radar diagram depicting average availability of facilities for all indicators in five domains (Human resource, Equipment, Laboratory service, Point-of-care supplies and Drugs). CHC- Community Health Centre; PHC- Primary Health Centre

Overall availability of facilities was lowest in the domains of laboratory services and human resource. Availability of all drug classes required for management of diabetes, hypertension and their acute complications was intermediate. Equipment and point of care supplies had better availability (Fig. 1). Availability of various facilities amongst CHCs of districts where NPCDCS is being piloted (*n* = 14) and other districts (*n* = 25) was similar.

In Human resource domain, while two medical officers were available in most CHCs, this was the case in less than half PHCs sampled. Similarly availability of Laboratory technicians and Health Educators was also significantly lower. Nutritionists and physiotherapists were mostly not available at either level of care (Table 2). In equipment domain, sphygmomanometer was available at all PHCs and CHCs, measuring tape was available in about 80 % of facilities, and height measurement equipment was available in about half of all facilities. Glucometer was available in most CHCs, but in less than half of the PHCs. ECG machine was available in only about one-third of CHCs, and less than 10 % of PHCs (Table 2). While point of care test supplies (blood glucose estimation strips/glucostrips, needles, urinary protein strips etc.) were available in most CHCs, their availability was significantly lower in PHCs. Availability of urinary ketone strip, useful for diagnosis of diabetes related complication, was available in only half of all CHCs, and a third of all PHCs. Availability of laboratory based tests was negligible at PHCs, and low at CHCs (Table 2).

**Table 2:** Availability of key items in community health centers and primary health centers

		Community health center			Primary health center		
Group	Variable	Available	Total	Percent	Available	Total	Percent
Variables assessed in full version of questionnaire (*n* = 85)							
Human resource	Laboratory technician	35	38	92.11	12	47	25.53
	Health Educator	17	38	44.74	10	47	21.28
Equipment	Glucometer	32	38	84.21	20	47	42.55
	Sphygmomanometer	38	38	100.00	47	47	100.00
	ECG machine	13	38	34.21	5	47	10.64
	Measuring tape	30	38	78.95	38	47	80.85
	Stadiometer and Weight machine	23	38	60.53	23	47	48.94
Point-of-care testing supplies	Glucostrips	33	38	86.84	24	47	51.06
	Lancets/needle	35	38	92.11	34	47	72.34
	Urinary ketone strip	22	38	57.89	17	47	36.17
	Urinary protein strip	31	38	81.58	27	47	57.45
Laboratory test	Plasma Glucose estimation	29	38	76.32	13	47	27.66
	Serum creatinine	17	38	44.74	4	47	8.51
	Serum lipid profile	10	38	26.32	4	47	8.51
	HbA1C	2	38	5.26	2	47	4.26
Additional variables assessed in second version of questionnaire (*n* = 37)							
Human resource	Two Medical Officers	10	12	83.33	11	25	44.00
	Nutritionist	2	12	16.67	0	25	0.00
	Physiotherapist	1	12	8.33	0	25	0.00
Equipment	Percussion hammer/tuning fork	6	12	50.00	12	25	48.00
Drugs	Any anti-hypertensive	12	12	100.00	22	24	91.67
	ACEI/ARB	6	12	50.00	7	25	28.00
	Beta blocker	12	12	100.00	17	25	68.00
	Calcium channel blocker (CCB)	11	12	91.67	21	25	84.00
	Diuretic Thiazide	4	12	33.33	4	25	16.00
	Atorvastatin	2	12	16.67	4	25	16.00
	Any anti-diabetic drug	11	12	91.67	17	25	68.00
	Metformin	10	12	83.33	17	25	68.00
	Glibenclamide	2	12	16.67	4	25	16.00
	Glimepiride	2	12	16.67	5	25	20.00
	Pioglitazone	1	12	8.33	2	25	8.00
	Insulin	5	12	41.67	3	25	12.00
	Aspirin low dose	6	12	50.00	11	25	44.00
	Frusemide	10	12	83.33	19	25	76.00
	Sorbitrate	9	12	75.00	12	25	48.00
	Nifedepine	10	12	83.33	17	25	68.00
	Dexamethasone	11	12	91.67	23	25	92.00

Among facilities for primary care management of hypertension, equipment for diagnosis and at least one anti-hypertensive drug was available at most PHCs and CHCs. In contrast, facility to screen for diabetes mellitus with help of glucometer was reported to be not available at more than half of PHCs and about a fifth of all CHCs. Availability of supplies or services to screen for complications related to these conditions was much lower (Table 2). Availability of drugs used in long-term treatment of hypertension, diabetes mellitus, and their acute complications was enquired from a subset of medical officers. Two of the four basic drugs needed in management of hypertension (beta-blockers and calcium channel blockers) were available at most PHCs and CHCs. One of four drugs for management of diabetes mellitus (metformin) was available at most CHCs and about two-thirds of all PHCs. Other drugs used in management of diabetes mellitus (other oral drugs and insulin) were largely unavailable (Table 2).

Low dose aspirin, which is most useful and inexpensive of all cardiovascular drugs was available at about half of all facilities. Lipid lowering drugs were available in less than one-sixth of all facilities. Nitrates were available at about three-fourths of all CHCs, and less than half of all PHCs. On the contrary, dexamethasone, a drug which is frequently misused in emergency management of cardiovascular complications was available at most PHCs and CHCs.

## Discussion

Current facility assessment study shows critical gaps in key items required for management of NCDs at primary care level. Human resource and laboratory services need to be strengthened the most, followed by sustained availability of all required drug classes, equipment and related supplies, and upgrading point-of-care testing. There are larger gaps in PHCs, which are level 1 facilities, as compared to CHCs, which are level 2 facilities in primary-care.

In order to achieve Universal health-care, there is a need to strengthen primary-care delivery in India. Deficiencies in human resources and laboratory services assessed as part of this study impact a wide range of health services beyond CVDs. While we have strengthened a wide range of specific services in primary-care in India (such as immunization coverage [12], availability of contraceptives [13], treatment of tuberculosis [14], institutionalized child-births [15]). As it is expected that 70 % of the health budget needs to be earmarked for primary health care under universal health coverage [16], we need to understand chain of CVD care resources (human resource, drugs, and health technology (equipment and laboratory services) that needs to be optimized to specifically improve CVD coverage in India.

First component in this chain are human resources. Management of CVDs like hypertension and diabetes requires life-long behavioral change which needs to be implemented using trained human resources. A PHC provides coverage to at least 30,000 population of which approximately 18,000 would be having age more than 15 years, which could mean about 3600 adults with hypertension, and about 600 with diabetes mellitus considering prevalence of hypertension and diabetes to be 20 and 3.3 % respectively. Even if a third of them were to seek care in the PHCs, medical doctors alone will never be sufficient to meet all the CVD-care needs. A medical doctor is considered necessary for first diagnosis and subsequent follow-ups, various task-components need to be shifted to supervised non-physician health workers. For instance, screening for hypertension and diabetes mellitus using blood pressure equipment or glucometers can be done with help of trained health-workers [17]. All individuals with hypertension and diabetes need intensive health education, meaningful dietary advice, and physical activity advice, reinforced at every follow up visit. This can be achieved by training existing multi-purpose community health workers, as it will not be feasible to employ dedicated nutritionists and physiotherapists.

Second component in CVD-care chain are drugs. Drug therapy in CVD is complex due to multiple drug categories, and multiple drugs in each category. More recent disease manegement guidelines have simplified drug therapy protocols. Core list of medicines in WHO PEN, required for implementing essential NCD interventions includes four anti-hypertensives (ACEI, beta-blockers, CCBs, and thaizide diuretic), three anti-diabetics (metformin, glibenclamide, insulin), aspirin, nitrates, frusemide and a lipid-lowering drug. Current study shows discordance in availability of recommended class drugs. For example, for hypertension beta blockers and CCBs are widely available but ACEI or Thiazide diuretcics are not. Further, we found that availability of Aspirin and Lipid lowering drugs was low, despite both of these drugs being useful in prevention of vascular events in individuals who have diabetes mellitus (primary prevention) or in those who already have had a previous event (secondary prevention). This indicates lack of standard protocols, poor convergence and patchy coordination between service providers and health system managers. This could be rectified by consultative group meeting of experts on subject including service providers. It also calls for revision in the essential drug lists of the State to keep pace with the changing guidelines in management of CVDs.

Third component in CVD-care chain is technology, which includes simple essential tools, and more complex laboratory support. List of simple essential tools in WHO PEN include a blood pressure measurement device, measuring tape, weighing machine, glucometer, urinary protein and ketone testing strips. Except for blood pressure measurement device, these simple technologies were deficient in many primary-care facilities of Madhya Pradesh. Additional complex technologies to be added when resources permit include facility to measure serum creatinine, lipids, and electrocardiography that were mostly not available. Average availability of these necessary drugs and technologies reported in the enrolled health-centers ranged between 11 and 55 % in PHCs, and 37 and 80 % in CHCs.

A similar study was done by Mendis et al. [18] in 90 facilities across eight low-and-middle-income countries, which reported similar deficiencies in human resources, drugs and technologies. Major deficits were identified in health financing, access to basic technologies and medicines, medical information systems, and the health workforce [18]. The growing burden of CVDs needs to be countered by strengthening primary care facilities, especially for hypertension and diabetes [19].

In current study, we were able to perform facility assessment with minimal resources, and in a short period of time. Since it was conducted by means of questionnaire administration, directed towards workshop participants, it has some inherent limitations. First limitation is selection bias as primary health centers were conveniently sampled based on medical officers posted in these PHCs who attended the workshop. This bias is likely to oversample those PHCs which have better facilities, as centers which did not have a medical officer to spare at the time will not be sampled. PHCs with such human resource deficit are more likely to lack in facilities. Despite this limitation our study samples facilities from half of all districts, and covers entire geographic spread of Madhya Pradesh. Second limitation is information bias, as we did not perform onsite assessment, and responses were not verifiable and therefore functionality of equipment could not be commented. However, we believe that medical officers, are more likely to over-report availability of certain key items. This is especially true about perishable items such as drugs. Last is measurement bias, as questionnaire based surveys such as ours report facilities which are generally present, rather than those present on a particular day or time. This bias is also likely to be away from null, and over-report availability of facilities. Despite these limitations, which are all operationally away from null, we report critical gaps in infrastructure which need to be addressed to make NCD control successful in public health facilities focused towards primary care.

## Conclusions

Preparedness for comprehensive care from prevention, early diagnosis to treatment and complication management of NCDs at primary care level is sub-optimal due to critical gaps in health facilities of Madhya Pradesh. These gaps highlight need for strengthening of primary health care systems for scaling up of interventions for prevention and management of chronic diseases so as to achieve goal of universal coverage and reduce the burden of NCDs.

## Abbreviations

ACEI: Angiotensinin Converting Enzyme Inhibitors
ARB: Angiotensin Receptor Blockers
CCB: Calcium Channel Blockers
CHC: Community Health Centre
CVD: Cardiovascular Diseases
IHEC: Institutional Human Ethics Committee
IPHS: Indian Public Health Standards
NCD: Non-Communicable Diseases
NPCDCS: National Program for Prevention & Control of Cancer, Diabetes, Cardiovascular Diseases and Stroke
PHC: Primary Health Centre
WHO PEN: World Health Organization -Package of Essential NCD interventions
